# Effectiveness and safety of tetanus vaccine administration by intramuscular vs. subcutaneous route in anticoagulated patients: Randomized clinical trial in primary care

**DOI:** 10.3389/fmed.2022.1054988

**Published:** 2022-12-22

**Authors:** Fernando Isidro Lago-Deibe, Mercedes Valladares-Cabaleiro, María José Fernández-Domínguez, Isabel Fernández-Fernández, Ana Clavería, Sara Rodríguez-Pastoriza, Javier Roca-Pardinas, María Victoria Martín-Miguel

**Affiliations:** ^1^Sárdoma Health Center, Vigo Health Area, Galician Health Service, Vigo, Spain; ^2^South Galicia Health Research Institute (Instituto de Investigación Sanitaria Galicia Sur), Vigo Health Area, Galician Health Service, Vigo, Spain; ^3^Network for Research on Chronicity, Primary Care and Health Promotion (Red de Investigación en Cronicidad, Atención Primaria y Promoción de la Salud/RICAPPS), Vigo, Spain; ^4^Moaña Primary Care Emergency Center (Punto de Atención Continuada), Vigo Health Area, Galician Health Service, Moaña, Spain; ^5^Leiro Health Center, Ourense Health Area, Galician Health Service, Ourense, Spain; ^6^Redondela Health Center, Vigo Health Area, Galician Health Service, Redondela, Spain; ^7^Department of Statistics and Operations Research, University of Vigo, Vigo, Spain; ^8^Galician Research and Mathematical Technology Center (Centro de Investigación e Tecnoloxía Matemática de Galicia/CITMAga), Santiago de Compostela, Spain; ^9^Vigo Family and Community Medicine and Nursing Teaching Unit, Vigo Health Area, Galician Health Service, Vigo, Spain

**Keywords:** vaccines, tetanus antitoxin/administration and dosage, drug administration routes, primary health care, clinical trial

## Abstract

**Design:**

Prospective, double-blind clinical trial comparing tetanus-diphtheria vaccine administration routes, intramuscular (IM) vs. subcutaneous (SC) injection, in patients with oral anticoagulants. ISRCTN69942081.

**Study population:**

Patients treated with oral anticoagulants, 15 health centers, Vigo (Spain). Sample size, 117 in each group.

**Outcome variables:**

Safety analysis: systemic reactions and, at the vaccine administration site, erythematic, swelling, hematoma, granuloma, pain.

Effectiveness analysis: differences in tetanus toxoid antibody titers.

Independent variables: route, sex, age, baseline serology, number of doses administered.

**Analysis:**

Following the CONSORT guidelines, we performed an intention-to-treat analysis. We conducted a descriptive study of the variables included in both groups (117 in each group) and a bivariate analysis. Fewer than 5% of missing values. Imputation in baseline and final serology with the median was performed. Lost values were assumed to be values missing at random. We conducted a descriptive study of the variables and compared routes. For safety, multivariate logistic regression was applied, with each safety criterion as outcome and the independent variables. Odds ratios (ORs) were calculated. For effectiveness, a generalized additive mixed model, with the difference between final and initial antibody titers as outcome. Due to the bimodal distribution of the outcome, the normal mixture fitting with gamlssMX was used. All statistical analyses were performed with the gamlss.mx and texreg packages of the R free software environment.

**Results:**

A previously published protocol was used across the 6-year study period. The breakdown by sex and route showed: 102 women and 132 men; and 117 IM and 117 SC, with one dose administered in over 80% of participants. There were no differences between groups in any independent variable. The second and third doses administered were not analyzed, due to the low number of cases. In terms of safety, there were no severe general reactions. Locally, significant adjusted differences were observed: in pain, by sex (male, OR: 0.39) and route (SC, OR: 0.55); in erythema, by sex (male, OR: 0.34) and route (SC, OR: 5.21); and in swelling, by sex (male, OR: 0.37) and route (SC, OR: 2.75). In terms of effectiveness, the model selected was the one adjusted for baseline serology.

## Introduction

While tetanus is an infrequent disease in Spain, it is nevertheless an important public health problem because, despite its low incidence, the related mortality is very high. Annual case reports show a gradual fall in numbers, with a total of 136 cases being reported in Spain across the period 2009–2015 (a mean of 10 cases per year), 25 of which proved lethal (18.4%) (1).

Tetanus is a disease that can be totally controlled, being preventable by vaccination, but it is not eradicable, since Clostridium tetani is a widespread microorganism found in the environment (2). Immunization is highly effective, affording long-term protection and is recommended for the general population, though for immunity to be maintained, a booster dose must be administered after completion of the primary vaccination (3).

Most tetanus cases occur in adults who have not previously been vaccinated, particularly in those over the age of 64 years. Of the cases reported in Spain from 2009 through 2015, 73.5% had not been vaccinated and 25.3% had received only one dose (1).

The 2017–2018 seroprevalence study in Spain showed that immunity against tetanus exceeds 90% at ages 6–49 years. As from age 50 years upwards, there is a gradual percentage increase in the susceptible population, falling to 40% immunity in the 70–80 age group (4). There are studies specifically targeting the elderly, which report a seroprevalence of 7.7% in persons over the age of 70 years (5, 6). The fact that the vaccination schedule with 5 tetanus doses was not introduced until the early 1970s in Spain probably accounts for the current situation, in which where the majority of adults aged over 50 years are either unvaccinated or incompletely vaccinated.

In the case of injuries, the need to administer active immunization (tetanus toxoid), whether alone or in tandem with passive immunization (anti-tetanus immunoglobulin), depends on the type of wound involved, the probability of its becoming contaminated with tetanus bacilli, and the patient's vaccine record (3). Currently, administration of the combined tetanus-diphtheria (Td) vaccine (presentation for adults) by intramuscular route is recommended (3).

Most patients who are anticoagulated in primary care settings and are being treated for atrial fibrillation have a mean age of over 74 years (7), which means in turn that their vaccine coverage is probably low (8). In anticoagulated patients, the use of the intramuscular (IM) route is usually not advised, due to the hypothetical risk of bleeding after puncture, with the result that the subcutaneous (SC) route is recommended, even for vaccines routinely administered by the IM route, such as the tetanus vaccine (9). It has to be said that sporadic cases of serious hemorrhagic complications are indeed reported in the literature (10, 11).

Even so, the vaccine efficacy studies were conducted using the IM route (9, 12, 13), and the SC route would be less effective than the IM route (14), though there is no uniformity of results in studies that compare the effectiveness of the two routes (13, 15, 16). Furthermore, for most vaccines, local reactions are more frequent with SC than with IM administration (15, 17, 18), though, aside from the route *per se*, needle size can also have an influence (19, 20).

With respect to hepatitis B (21) and influenza vaccines (22, 23), where IM administration is concerned, this route's safety has been demonstrated in patients with coagulation alterations, and as a consequence, the 2006 CDC guideline recommends the IM route for Td vaccine, subject in every case to the physician's judgment. There is a systematic review that compares the efficacy of the IM and intradermal routes in influenza vaccine (24), but we were unable to find any study in the literature that assessed the safety and efficacy of the IM and SC routes for the Td vaccine in patients receiving oral anticoagulant therapy (OAT).

Hence, the aim of this study was to compare the safety and efficacy of the IM and SC administration routes for the Td vaccine in OAT patients, in order to test the hypothesis that the IM route is safer and more efficacious.

## Materials and methods

### Trial design

Prospective, double blind clinical trial, in which two groups of patients treated with oral anticoagulants were compared. Each group received doses of tetanus vaccine by a different route, IM or SC.

The protocol was registered in www.isrctn.com under number ISRCTN69942081 and published (25), in accordance with the CONSORT 2010 guidelines (26).

### Participants

Patients on monitored treatment with oral anticoagulants at 15 health centers in the Vigo Primary Care Area.

- Inclusion criteria: Patients indicated to receive at least one dose of tetanus vaccine, and treated with anticoagulants. This criterion was applied to those whose vaccination record was unknown or uncertain, or had not been vaccinated. Persons who gave informed written consent to receive the vaccine and participate in the study.
- Exclusion criteria: Severe local reaction to previous doses, affecting the entire area of the extremity where the vaccine had been injected. Peripheral neurologic disorders caused by previous doses. Severe anaphylactic reaction due to previous doses or any of their components. Poor hematologic control in the preceding 2 months. Persons with terminal disease states, severe illness, adversely affected by chronic pathology, immobilized, or in an immunosuppressive state. Pregnant or lactating women.

At the time of the study, the medication allowed by the public health system was acenocoumarol. In accordance with the organization's protocols, the INR was used to verify adequate anticoagulation control. Patients with coagulation disorders were not followed up in primary care and were not included in the study, and those with INR greater than 4 were not included until after 1 month in range.

This trial was purpose-designed for the study of vaccines in anticoagulated populations, but not for wound scenarios.

### Interventions

All the patients included in the study were anticoagulated with Acenocoumarol (Sintrom^®^). Patients were recruited by their family physicians (FPs) at primary care health centers. At the first visit, the physicians determined the patient's vaccination status, by consulting the vaccination records in the patient's medical history or asking the patient in cases where this information was unknown. The FP could then evaluate whether the patient had been adequately vaccinated (in which case, he/she was excluded from the study), or alternatively, whether the patient needed to receive a booster dose, or initiate or complete his/her primary vaccination. The doctor explained the study and requested the patient's consent. Once it was signed, the patient was attended by the nurse, who proceeded to perform the INR, the serology extraction and the entire procedure as established in the protocol. The guidelines used for the pertinent vaccinations were those issued by the Spanish Ministry of Health in 2008 (27).

Recruitment started in January 2009, with an initial forecast of 24 months' participation. The monitoring for each dose was undertaken at 24 h, at 48 h, at 15 and 30 days following the inoculation of the vaccine. The patients were cited on Monday, Tuesday, and Wednesday. None of them were cited during the weekend in local or national holidays.

Data were recoded on a purpose-designed case report form (CRF). In addition, a specific database was created for uploading and storing the observations collected.

Prior to the study, a training workshop was held for all researchers participating in the clinical trial, covering techniques, data-collection, and the measurement of study variables.

### Outcomes

The main outcome variables for safety were:

- Appearance of baseline lesions at the site where the vaccine was administered (redness, swelling, heat, granuloma, hematoma), axillary lymph nodes, and pain as scored on a visual analog scale (28), consisting of a 10-centimeter-long horizontal line ranging end-to-end from the lowest to the highest extremes of pain. Measurement of the brachial perimeter in centimeters;
- Appearance of general symptoms (fever, general malaise, headache, weakness, arthralgias); and,
- Occurrence of any serious adverse effect, fatal or life-threatening to the patient, resulting in incapacity or requiring hospitalization.

The difference between tetanus antibodies at baseline and post-vaccination was the main outcome variable in the effectiveness analysis. Tetanus antitoxin was analyzed by enzyme immunoassay at a centralized laboratory.

As independent variables, route, sex, and age were considered. Initial International Normalized Ratio (INR) were determined by capillary reflectometer technique for clinical control purposes.

### Sample size

Assuming that the percentage of local side effects for the IM route was 30%, and that the expected increase in local side effects for the alternative (SC) route using a bilateral approach with a 95% confidence interval was 18%, and a beta risk of 0.20, we calculated that 115 patients for each group would be required. In view of potential data losses of 15%, the final sample size was set at 135 patients for each group. Based on this sample size, we estimated that a mean difference of 3 IU/ml in antibody levels could be detected.

### Randomization

The unit of randomization comprised the individuals participating in the trial. Randomization was performed within a 3-level stratification, based on the number of vaccine doses required for successful immunization. Within each level, simple randomization was performed, using a spreadsheet in an attempt to control for the confounding effect of the number of doses. Sampling was performed by the Fundación Galicia Sur (EOXI Vigo), to which all researchers were given telephone access.

Data relating to the randomization process were kept confidential until the end of the study. It was each researcher's responsibility to ensure that there was a specific procedure which enabled the code to be opened in case of emergency, with immediate notification of this to the randomization center.

The vaccine route was masked in the dataset, to prevent the team tasked with the computer analysis from discovering which route corresponded to each group. Loss masking only occurred in cases of patient emergency and at the end of the study.

### Blinding

The physician was blinded to the original administration route at the control visit to detect side effects.

### Quality control

In the case of patients with whom contact and follow-up was lost, the researcher documented the steps taken to recontact them. Data contained in each patient's CRF were periodically checked and reviewed, to ensure that they were complete and regularly updated, and that the good clinical practices required by the protocol and follow-up procedures were being maintained. The researchers kept the original documents for each patient who participated in the study, consisting of the notes made at each visit and the original signed informed consent forms.

### Statistical methods

Quantitative data were summarized with the arithmetic mean and standard deviation for parametric variables, and with the median, 25th percentile, and 75th percentile otherwise. For qualitative variables, number and proportions were applied.

Considering an alpha error of 0.05, and after checking the necessary conditions, chi-square or Fisher tests were applied to determine the statistical differences between qualitative variables among groups. The t-Student test or Mann-Whitney *U*-test were used to compare quantitative variables.

Intention-to-treat (ITT) analysis was performed in accordance with the CONSORT guidelines (26).

First, we performed a multivariate logistic regression, considering the dichotomous safety variables—pain, erythema, swelling, hematoma, and granuloma—as the target, and adjusting for the independent variables (sex, age, route). Odds ratios (ORs) and their confidence intervals were then calculated. We also built suitable generalized additive models for position, scale, and shape, to explain the adjusted outcome for effectiveness (differences in antibody level). Initial serology was considered as independent variable.

All analyses were performed using the R Studio statistical software package version 4.1.3. (29).

### Ethical aspects and data confidentiality

This study obtained authorization from the Galician Clinical Research Ethics Committee on 07/06/2007 under number 2007/089 (No. EudraCT 2007-001073-29). This authorization was subsequently modified and extended on 13/04/2009, 10/09/2009, and 13/12/2010 to increase patient enrollment.

## Results

### Participant flow

Of the 375 individuals initially considered for participation in the study, 234 were included and 141 declined to participate in the study or were not eligible.

**Figure 1:**
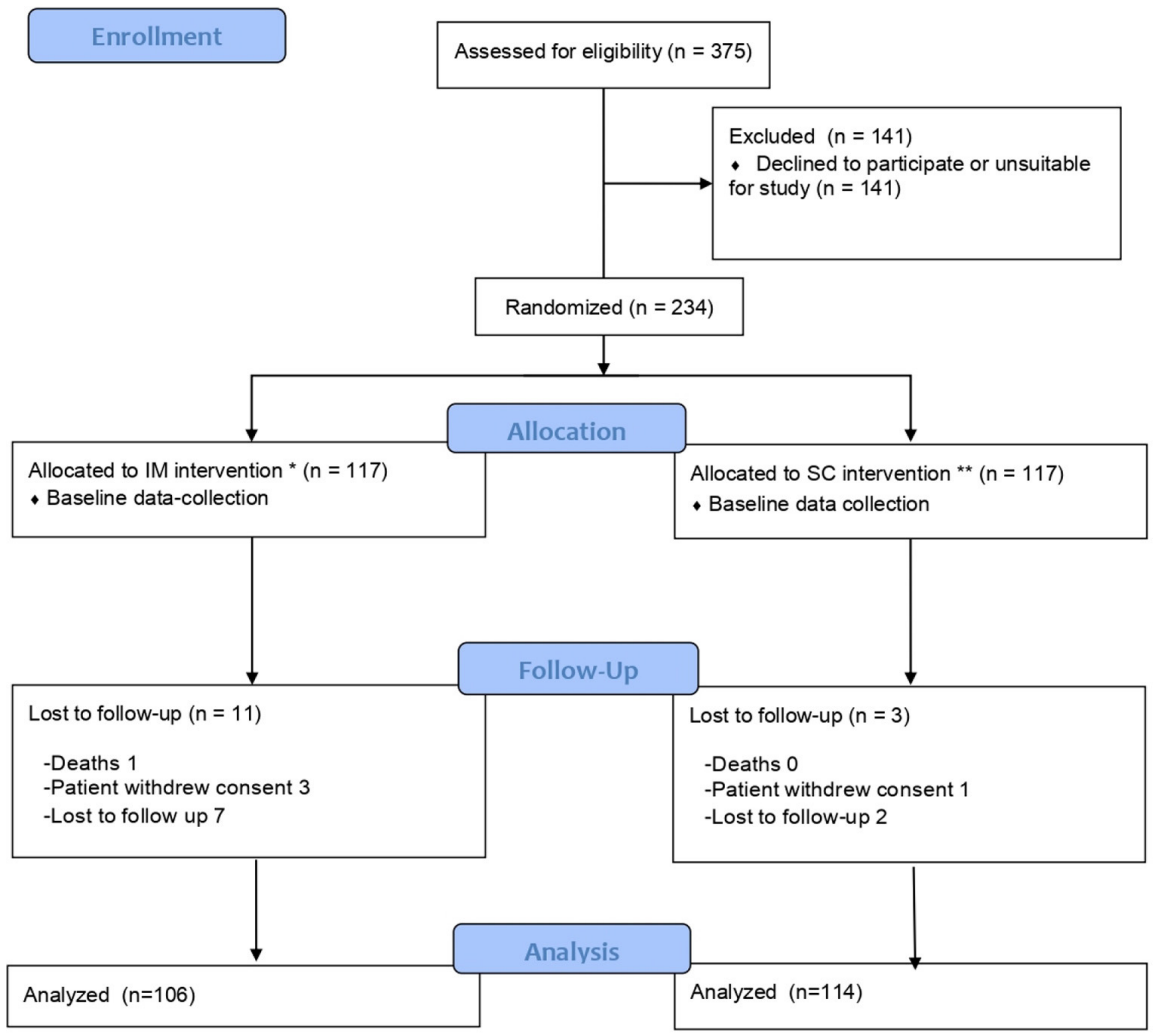
Flow chart of study methodology.

The 234 participants were randomized into two groups, by type of route: 117 were vaccinated by the IM route and 117 by the SC route, and of these, 106 and 114 were, respectively, included for study purposes. The flow chart is shown in Figure 1.

### Recruitment and follow-up

Recruitment was subsequently extended to 72 months, due to difficulties encountered in achieving the predetermined sample size.

The causes for patients' premature abandonment were side effects in the vaccination process, withdrawal of consent, loss to follow-up, administrative problems, and death.

### Baseline data

A breakdown of the baseline quantitative variables showed that: the initial tetanus antitoxin titers had a median value of 531 (25th percentile, 31; 75th percentile, 1226); the median age was 73 years (25th percentile, 67; 75th percentile, 79); and the required dose variable had a median of 1 dose (interquartile range, IQR: 1;1). When it came to the baseline qualitative variables, 50% of the individuals were vaccinated by the SC route, and 56.41% were men.

A bivariate analysis was performed, with the non-parametric Mann–Whitney U and chi-square tests being used to ascertain the existence of significant differences between the two groups (Table 1). The same number of patients were allocated to each route. In the final tetanus antitoxin titration, nine patients were lost to follow-up. None of the independent variables showed any significant difference between routes. INRs had significant differences, but they are not clinically relevant.

**Table 1:** Bivariate analysis by route.

**Route**	**Intramuscular^#^**		**Subcutaneous^#^**		***p***
		**NA**		**NA**	
Age	73.00 (67/79)		73.62 (66/80)		0.930
Sex					0.895
Women	50 (42.74)		52 (44.44)		
Men	67 (57.26)		65 (55.66)		
INR basal	2.40 (1.9/2.8)	7	2.50 (2.1/2.8)	4	0.000^***^
Initial serology	531.00 (30/1025)	4	531.00 (71/1276)	1	0.363
Required dose					0.388
1	88 (75.21)		94 (80.34)		
2	10 (8.55)		5 (4.27)		
3	19 (16.24)		18 (15.38)		

# Data are expressed as median (*P*_25_/*P*_75_) or absolute frecuency (%).

*** *p* < 0.001; ^**^p < 0.01; ^*^p < 0.05.

### Outcomes and estimates

In the IM group, 80% of participants had previously received one dose, 8% had received two doses, and 13% had received three doses: in the SC group, 82% of the participants had previously received one dose, 6% had received two doses, and 1% had received three doses.

Pain was recoded into a dichotomous variable (1:Yes; 0:No), with “Yes” defined as any scale score greater than zero. The other elementary lesions (erythema, swelling, hematoma and granuloma) at the vaccine-administration site were recoded as dichotomous variables (1: Yes; 0: No), with any value greater than 0 mm. being classified as “Yes”.

Shown in the Annex (Supplementary material) are the results of the bivariate analysis (performed as per the protocol) by route, with 106 vaccinated by the IM route and 114 by the SC route.

To reduce any bias due to lack of data during follow-up, an ITT analysis was performed, with the missing values of the initial and final tetanus antitoxin titer variables being imputed *via* their medians.

The following variables displayed significant differences for dichotomous outcomes: age, with swelling; sex, with erythema and swelling; and route, with erythema and swelling (Table 2).

**Table 2:** Bivariate analysis of outcome variables.

**Route**			
	**Intramuscular** ^#^	**Subcutaneous** ^#^	***p***
Pain (Yes)	45 (58.44)	32 (41.56)	0.074
Erythema (Yes)	5 (18.52)	22 (81.48)	0.001^**^
Swelling (Yes)	9 (29.03)	22 (70.97)	0.023^*^
Hematoma (Yes)	2 (33.33)	4 (66.67)	0.695
Granuloma (Yes)	1 (20.00)	4 (80.00)	0.377
Final serology	4214.00 (1978/5001)	4260.00 (1956/5001)	0.803

# Data are expressed as median (*P*_25_/*P*_75_) or absolute frequency (%).

^***^p < 0.001;

** *p* < 0.01;

* *p* < 0.05.

The safety analysis showed that while no severe side effects were in evidence, there were general reactions, such as fever, headache, and arthralgia in the two routes under study (3.42% by the IM route, and 0.85% by the SC route).

To adjust for independent variables, multivariate logistic regression models were built. Their estimates of the coefficients are shown in Table 3. These models were built with the *glm* function of the *stats* package (30), applying the *step* function of R to select the significant variables. The independent variables considered were age, sex, route, and initial tetanus antitoxin titration.

**Table 3:** Multivariate logistic regression with pain, erythema, swelling, hematoma, and granuloma considered as the targets.

	**Pain**	**Erythema**	**Swelling**	**Hematoma**	**Granuloma**
	**outcome**	**outcome**	**outcome**	**outcome**	**outcome**
Intercept	0.15	−2.56^***^	−1.96^***^	−3.59^***^	−3.87^***^
	(0.25)	(0.48)	(0.38)	(0.41)	(1.05)
Route (Subcutaneous)	−0.60^*^	1.65^**^	1.01^*^		1.46
	(0.29)	(0.52)	(0.43)		(1.13)
Sex (Male)	−0.95^**^	−1.09^*^	−0.99^*^		
	(0.29)	(0.44)	(0.41)		
Initial serology					−0.00
					(0.00)
AIC	279.71	151.79	173.78	57.28	47.35
BIC	289.95	162.02	184.01	60.69	57.58
Log likelihood	−136.86	−72.89	−83.89	−27.64	−20.67
Deviance	273.71	145.79	167.78	55.28	41.35
Num. obs.	224	224	224	224	224

*** *p* < 0.001;

** *p* < 0.01;

* *p* < 0.05.

AIC, Akaike information criterion; BIC, Bayesian information criterion.

Lastly, the exponential transformations of the estimates of the coefficients associated with the qualitative variables were obtained, in order to ascertain the ORs, which are shown in Table 4 below, along with their corresponding confidence intervals.

**Table 4:** OR coefficients and confidence intervals for qualitative predictor variables.

	**Pain**	**Erythema**	**Swelling**	**Granuloma**
	**outcome**	**outcome**	**outcome**	**outcome**
	**OR (CI)**	**OR (CI)**	**OR (CI)**	**OR (CI)**
Route (Subcut.)	0.55 (0.31, 0.97)^*^	5.21 (2.01, 16.14)^**^	2.75 (1.22, 6.62)^*^	4.31 (0.62, 85.78)
Sex (male)	0.39 (0.22, 0.68)^**^	0.34 (0.13, 0.79)^*^	0.37 (0.16, 0.82)^*^	

^***^p < 0.001;

** *p* < 0.01;

* *p* < 0.05.

The following independent variables proved to be significant for the respective outcomes in the multivariate logistic model. In the case of pain, route (0.55, CI: 0.31, 0.97) and sex were significant (0.39, CI: 0.22, 0.68): patients who were male and had been subcutaneously vaccinated were less likely to experience pain. For erythema, the independent variables that were significant were route (5.21, CI:2.01, 16.14) and sex (0.34, CI: 0.13, 0.79): there was a 5.19 times greater chance of developing erythema when vaccines were given subcutaneously, and women were more likely to develop erythema than were men. For swelling, the independent variables that proved to be significant were route (2.75, CI: 1.22, 6.62) and sex (0.37; CI: 0.16, 0.82): patients were 2.74 times more likely to experience swelling, if the vaccine was given subcutaneously, and women were more likely than men to experience swelling. The predictor variable that was significant in the case of granuloma was route (4.31, CI: 0.62, 85.78).

**Figure 2:**
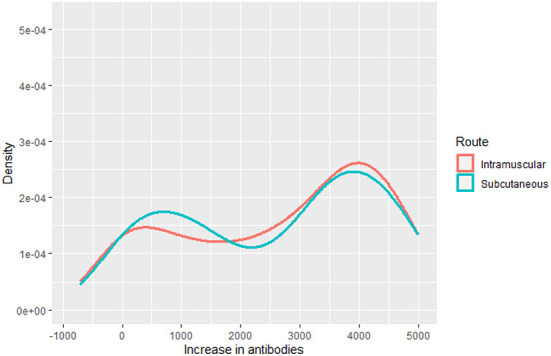
Density functions of increase in antibodies by route.

To model the final tetanus antitoxin titration, we considered the increase in antibodies, constructed from the difference between final and baseline tetanus antibodies. We used a multivariate regression model, selected after analyzing the distribution of the outcome variable (Figure 2).

In our case, the chosen distribution was a finite mixture of normal distributions and we thus analyzed it with the gamlss.mx package (31). The parameters of the distribution of the outcome were modeled as functions of the independent variables, i.e., age, sex, route, and initial tetanus antitoxin titers.

After analyzing the behavior of several models by varying the different independent variables, we compared these according to the AIC criteria. The model chosen with the lowest AIC was thus the one that considered the baseline serology variable as a significant influential variable. A detailed summary is shown in Table 5 below.

**Table 5:** Generalized additive model of normal mixture distribution where the dependent variable is the increase in antibodies.

**Mixing Family: c("NO". "NO")**
Fitting method: EM algorithm
Call: gamlssMX(formula = dif ~sqrt(inisero). family = NO.
K = 2. data = datasetaux1. control = MX.control(plot = FALSE))
Mu Coefficients for model: 1
(Intercept) sqrt(inisero)
1289.208 -8.693
Sigma Coefficients for model: 1
(Intercept)
6.862
Mu Coefficients for model: 2
(Intercept) sqrt(inisero)
4716.84 -33.13
Sigma Coefficients for model: 2
(Intercept)
6.115
Estimated probabilities: 0.4577287 0.5422713
Degrees of Freedom for the fit: 7 Residual Deg. of Freedom 227
Global Deviance: 3974.63
AIC: 3988.63
SBC: 4012.82

AIC, Akaike information criterion; SBC, Schwarz Bayesian information criterion.

The behavior of the residuals associated with the chosen model was analyzed, and only about 5% of the observations were distributed outside the confidence intervals. Their representation is included in the Annex (Supplementary material), and indicate that the model is adequate.

## Discussion

This study was an independent, double-blind, randomized clinical trial (RCT), designed with the dual aim of comparing the effectiveness and safety of the SC and IM administration routes of DTP vaccine in anticoagulated adults. The outcome variables relate to the comparison of immunogenicity and reactogenicity between the two tetanus vaccine administration routes: SC vs. IM. With respect to safety, there was just one systemic reaction. Locally, there were significant adjusted differences in the following: pain, by sex (male, OR: 0.39) and route (SC, OR: 0.55); erythema, by sex (male, OR: 0.34) and route (SC, OR: 5.21); and swelling, by sex (male, OR: 0.37) and route (SC, OR: 2.75). In terms of effectiveness, the model selected was the one adjusted for baseline serology.

With respect to immunogenicity, this RCT found a comparable increase in antibody rates for the two routes, but without differences in terms of effectiveness. This finding is in line with other clinical studies published to date, which observe similar immune responses induced by SC and IM immunizations, in various types of vaccines, including: rabies (Kulkarni PS 2013); hepatitis A; hepatitis B (16); influenza; HIV (18); measles-mumps-rubella-varicella (MMRV); MMR; and meningococcal (32). Clinical trials on the pediatric population of the United Kingdom, Sweden and USA report comparable immunogenic responses for both administration routes, SC and IM, for Td and DTP vaccines (15, 20, 33). However, intradermal inoculation of hepatitis A, hepatitis B, rabies, influenza, and human papillomavirus vaccines, generates a greater immune response than does the IM route with equivalent doses, which range, according to studies, from 10 to 60% of that used by the IM route (32).

Apart from the administration route, other external factors are described which would potentially modify immunogenicity, such as the anatomic site of inoculation (34), technique and needle size (35, 36), type of adjuvant (37), interval between immunizations, vaccination strategy (32), and concomitant intake of drugs. Simultaneous administration of paracetamol would produce lower antitetanus antibody rates in children, which remain lower after a booster dose (38). Ingestion of ibuprofen causes a lower rate after the first dose, but this does not remain low after the booster (39). Stations, in the case of older adults, would lower the immune response to influenza vaccine but there are no studies on tetanus vaccine (40). Furthermore, the influence of intrinsic factors on the immune response to vaccination has been described, in that the elderly would have a lower response as well as a faster antibody clearance rate (6, 41, 42). Men would obtain a greater degree of seroprotection with the tetanus vaccine than would women (6, 43). There is a clear genetic influence on responses to vaccines, with an estimated degree of inheritability for tetanus of 44% (44). Another factor is the pre-existing level of immunity: individuals who have higher tetanus antibody titers prior to vaccination, have higher seroprotection rates after the booster vaccination (41). Among older adults, a positive state of mind at the time of inoculation would induce better responses, Ayling K 2018, whereas chronic stress would produce lower antibody responses (45).

With reference to safety, only general reactions were observed. Local effects, such as erythema and swelling, were significantly more pronounced with SC administration, with a *p*-value of 0.01 and 0.023, respectively. Other studies on the child population would be consistent with these findings, and describe greater reddening and swelling with administration of Td *via* the SC route as opposed to the IM route (15, 19, 33). Likewise, similar local reactions have been reported on comparing the two routes, SC vs. IM, in children with the MMR vaccine (Knuf M 2010), and in adult males with the influenza vaccine (32).

Furthermore, the IM route was found to cause more pain than the SC route, something that is plausible, bearing in mind that the number of nociceptive nerve endings is higher in the muscle than in the subcutaneous space (14). Even so, other studies state the contrary, reporting more pain with the SC route (15, 19).

In general, the SC route may be associated with local irritation, swelling and hardening, discoloration of the skin, inflammation, and formation of granulomas. IM administration is especially recommended for adjuvanted inactivated vaccines, such as tetanus (32). Needle size too would have an influence on the appearance of side effects for IM inoculations; the deepest injections would produce fewer local effects (19, 46).

In addition, this RCT found that women present with more side effects than do men, without it being possible to corroborate this by reference to the literature.

Clinical trials with the influenza vaccine in the anticoagulated population, which, like ours, compared the two administration routes, IM and SC, report findings similar to ours, in that they observe no differences in immunogenicity or reactogenicity (22, 23, 47).

This study was an independent, double-blind RCT, which was designed, managed and organized in primary care, and funded with the aid of a Regional Health Authority grant awarded in a competitive call for tender. In this context, the difficulties of conducting an RCT are greater than in a hospital. The obstacles were linked to limited funding, complex coordination owing to the wide dispersion and participation of a great number of researchers, increased complication in the logistics of clinical analysis and CRFs, and problems in adherence experienced by participating patients, mostly elderly dependents in a variety of circumstances (transport, accessibility, family situation, etc.).

At the time when the study began, control of anticoagulation was being implemented in primary care and there were not that many anticoagulated patients in the population (48, 49). That said, however, many of these patients did not meet the criteria for vaccination, while others either had no interest in participating in the study or depended on the support of family members, neighbors or friends, to attend the medical visits, something that made for a laborious recruitment process, which delayed the proposed sample size being achieved and, by extension, the study being brought to an end.

The measure of effectiveness in this RCT was verified by antibody titration, as in almost all RCTs designed to study the immunogenicity of vaccines. This can be considered a limitation, given that small variations in antibody concentrations between groups of persons may not be clinically relevant insofar as vaccine protection is concerned. What is important is the quality of response of the antibodies, since only one subgroup of all those detectable could neutralize pathogens, and, in addition, the innate, cellular and cytokine response would also mediate in the efficacy of vaccines (45). Yet the complex mechanisms and interactions of the immune system to the response of vaccines are not well-established, and there are no known markers for monitoring this. Furthermore, this study evaluated the difference in levels for each individual. Negative values were observed for the variable increase in antibodies: there were eight cases with a decreased amount of antibodies as compared to baseline, without any common characteristics in the variables analyzed. On average, the decrease in antibodies in these patients was found to be 199. This is possibly explained by personal variability in the immune system over time or, alternatively, by incidents in sample management and/or analytical determination.

One strength of this study is the undertaking of an RCT in primary care, since this is known to be the setting where RCTs assume greatest relevance, since it is the environment where most treatments are applied and where the effectiveness and iatrogenicity of therapies can be most realistically evaluated (50, 51). There is any widely accepted concept of effect size for GAMLSS. The complexity of GAMLSS makes the application of statistical tests less straightforward, and it is even more complicated with a bimodal distribution. Therefore, we included visualization tools, comparing the outcome in both groups (Figure 2).

Many authors contend that most vaccines should be administered by the IM route, given the fact that there is evidence of a high degree of reduced reactogenicity, and this optimizes immunogenicity (13, 14). Even so, the guideline requiring subcutaneous vaccination for special groups at high risk of hemorrhage remains in place, due to the danger of hematomas secondary to the injection (11). This study's contribution to the currently ongoing discussion and debate would be another strong point, though our results cannot be extrapolated to patients with congenital hemorrhagic disorders or to those treated with direct-acting oral anticoagulants (DOACs). In our setting, the regulations governing the use of DOACs are moderately restrictive and, for the present, this continues to be a small group of patients.

In light of our results, there are no administration-route-related differences in immunogenicity for the TDP vaccine, and the IM route may thus be indicated, given that the risk of hematoma is minimal in patients treated with vitamin K antagonists, which maintain anticoagulation levels in range. While the local reactogenicity of the SC route is higher, there is more pain with the IM injection, with variations by sex, thus making it advisable for patients to be brought into the decision-making process (42, 52).

## Data availability statement

The raw data supporting the conclusions of this article will be made available by the authors, without undue reservation.

## Ethics statement

The studies involving human participants were reviewed and approved by Clinical Research Ethics Committee of Galicia. The patients/participants provided their written informed consent to participate in this study.

## ECA-VAT-TAO group members and their affiliated health centers, Vigo Health Area, Galician Health Service

María Teresa Peteiro Rodríguez; A Guarda Health Center

JacintoMosquera Nogueira; Bembrive Health Center (Vigo)

Andrés Barreiro Prieto; Chapela Health Center

Carlos Benito Andrade Cochón; Moaña Health Center

Julia Bóveda Fontán; Pintor Colmeiro Health Center (Vigo)

María ángeles Charle Crespo; Porriño Health Center

Jesús Martínez Barrios; Redondela Health Center

Manuel Domínguez Sardiña; Sárdoma Health Center (Vigo)

Luis López Vilar; Teis Health Center (Vigo)

Reyes Macías Alonso; Tomiño Health Center

Susana Aldecoa Landesa; Beiramar Health Center (Vigo)

Félix Tojal del Casero; Doblada Health Center (Vigo)

Susana Hernáiz Valero; Val Miñor Health Center

José María Sàez de Biteri; Matamà Health Center (Vigo)

Isabel Rey Gómez-Serranillos; Xerencia de Atención Primaria de Vigo (Vigo)

Carmen Velicia Peñas; Sárdoma Health Center (Vigo)

Manuela Fontanillo Fontanillo; Fundación Biomédica del CHUVI (Vigo).

## Author contributions

FL-D and MM-M conceived, designed the study, and wrote the first draft of the manuscript. FL-D, MM-M, and MV-C organized the database. MV-C and IF-F coordinate the fieldwork. FL-D, MM-M, MV-C, and IF-F conducted the literature search. AC, JR-P, and SR-P performed the statistical analysis. FL-D, MD-F, IF-F, AC, and MM-M interpreted the results. All authors reviewed and approved the final manuscript.
