# Supplementary material for: Effectiveness and safety of tetanus vaccine administration by intramuscular vs. subcutaneous route in anticoagulated patients: Randomized clinical trial in primary care
Source: Front Med (Lausanne). 2022 Dec 22;9:1054988. doi: 10.3389/fmed.2022.1054988 (PMC9813590; doi:10.3389/fmed.2022.1054988)
Supplement: Supplementary file 1 [file Data_Sheet_1.PDF]

ANNEX

1. Table

Bivariate analysis by route depending to vaccine dose number					
			**Route**		
			**Intramuscular**	**Subcutánea**	
			Mean (SD) or absolute frecuency (%)	Mean (SD) or absolute frecuency (%)	p
**Baselines characteristics**					
Age (years)			72.19 (10.61)	72.58 (9.50)	
Gender	Females		39 (44.32)	38 (40.43)	0.792
	Males		49 (55.68)	56 (59.57)	
Basal antitoxoid tetanus antibodies (mUI/ml)			923.36 (1168)	1,055.63 (1,144.42)	
Serology index			53.06 (115.98)	40.53 (154.30)	
Distribution according to vaccine dose number	0 doses		1 (1.14)	1 (1.06)	0.388
	1 dose		87 (98.86)	92 (97.87)	
	2 doses		0 (0)	1 (1.06)	
	3 doses		0 (0)	0 (0)	
Basal systolic pressure			140.723 (19.09)	143.15 (17.97)	
Basal diastolic pressure			77.80 (12.51)	81.31 (11.90)	
Basal brachial perimeter (mm)			370.85 (59.96)	370.93 (69.35)	
Basal temperature			35.93 (0.46)	35.95 (0.61)	
Basal INR			2.36 (0.59)	2.51 (0.54)	
**Outcome measures**					
Final serology			3,531.11(1680.95)	3,541.04(1729.55)	
**First dose**					
Appearance of basic injuries in the area of administration of the vaccine (number of cases) 1 day follow up	Pain	No	51 (58.62)	67 (72.83)	0.054
		Yes	36 (41.38)	25 (27.17)	
	Erythema	No	83 (95.40)	78 (84.78)	0.001
		Yes	4 (4.60)	14 (15.22)	
	Swelling	No	80 (91.95)	77 (83.70)	0.014
		Yes	7 (8.05)	15 (16.30)	
	Hematoma	No	86 (98.85)	88 (95.65)	0.421
		Yes	1 (1.15)	4 (4.35)	
	Granuloma	No	86 (98.85)	88 (95.65)	0.181
		Yes	1 (1.15)	4 (4.35)	
	Heat	No	78 (89.66)	79 (85.87)	0.117
		Yes	9 (10.34)	13 (14.13)	

Emergence of general symptom (number of cases) 1 day follow up	Adenopathy	No	87 (100)	92 (100)	
		Yes	0 (0)	0 (0)	
	Fever	No	84 (96.55)	90 (97.83)	0.637
		Yes	3 (3.45)	2 (2.17)	
	Headache	No	83 (95.40)	91 (98.91)	0.168
		Yes	4 (4.60)	1 (1.09)	
	Arthralgias	No	86 (98.85)	92 (100)	0.312
		Yes	1 (1.15)	0(0)	
	Malaise	No	79 (90.80)	90 (97.83)	0.049
		Yes	8 (9.20)	2 (2.17)	
	Weakness	No	84 (96.55)	91 (98.91)	0.304
		Yes	3 (3.45)	1 (1.09)	
	Temperature		36.02 (0.62)	36.01 (0.58)	
Appearance of basic injuries in the area of administration of the vaccine (number of cases) 2 day follow up	Pain	No	60 (68.97)	70 (76.09)	0.328
		Yes	27 (31.03)	22 (23.91)	
	Erythema	No	79 (90.80)	75 (82.42)	0.024
		Yes	8 (9.20)	16 (17.58)	
	Swelling	No	79 (90.80)	73 (80.22)	0.012
		Yes	8 (9.20)	18 (19.78)	
	Hematoma	No	62 (100)	76 (97.44)	0.660
		Yes	0 (0)	2 (2.56)	
	Granuloma	No	86 (98.85)	88 (95.65)	
		Yes	1 (1.15)	4 (4.35)	
	Heat	No	76 (87.36)	80 (86.96)	0.345
		Yes	11 (12.64)	12 (13.04)	
Emergence of general symptom (number of cases) 2 days follow up	Adenopathy	No	87 (100)	92 (100)	
		Yes	0 (0)	0 (0)	
	Fever	No	84 (96.55)	88 (95.65)	0.719
		Yes	3 (3.45)	4 (4.35)	
	Headache	No	85 (97.70)	91 (98.91)	0.551
		Yes	2 (2.30)	1 (1.09)	
	Arthralgias	No	85 (97.70)	92(100)	0.152
		Yes	2 (2.30)	0 (0)	
	Malaise	No	81 (93.10)	91 (98.91)	0.300
		Yes	6 (6.90)	1 (1.09)	
	Weakness	No	83 (95.40)	92(100)	0.042
		Yes	4 (4.60)	0 (0)	
	Temperature		36.10 (0.67)	36.03 (0.61)	
Appearance of basic injuries in the area of administration of the vaccine (number of	Pain	No	77 (88.51)	86 (95.56)	0.037
		Yes	10 (11.49)	4 (4.44)	
	Erythema	No	86 (98.85)	90 (100)	0.316
		Yes	1 (1.15)	0 (0)	
	Swelling	No	86 (98.85)	89 (98.89)	0.313
		Yes	1 (1.15)	1 (1.11)	
	Hematoma	No	86 (98.85)	90 (100)	0.316
		Yes	1 (1.15)	0 (0)	

cases) 14 day follow up	Granuloma	No	62 (100)	76 (98.70)	0.350
		Yes	0 (0)	1 (1.30)	
	Heat	No	86 (98.85)	90 (100)	0.316
		Yes	1 (1.15)	0 (0)	
Emergence of general symptom (number of cases) 14 days follow up	Adenopathy	No	87 (100)	90 (100)	
		Yes	0 (0)	0 (0)	
	Fever	No	87 (100)	89 (98.89)	0.316
		Yes	0 (0)	1 (1.11)	
	Headache	No	87 (100)	88 (97.78)	0.561
		Yes	0 (0)	2 (2.22)	
	Arthralgias	No	87 (100)	89 (98.89)	0.316
		Yes	0 (0)	1 (1.11)	
	Malaise	No	87 (100)	90 (100)	
		Yes	0 (0)	0 (0)	
	Weakness	No	86 (98.85)	90 (100)	0.316
		Yes	1 (1.15)	0 (0)	
	Temperature		35.92 (0.49)	35.96 (0.57)	
Appearance of basic injuries in the area of administration of the vaccine (number of cases) 30 day follow up	Pain	No	86 (100)	90 (97.83)	
		Yes	0 (0)	2 (2.17)	
	Erythema	No	86 (100)	92 (100)	
		Yes	0 (0)	0 (0)	
	Swelling	No	86 (100)	92 (100)	
		Yes	0 (0)	0 (0)	
	Hematoma	No	86 (100)	92 (100)	
		Yes	0 (0)	0 (0)	
	Granuloma	No	86 (100)	91 (98.93)	
		Yes	0 (0)	0 (0)	
	Heat	No	86 (100)	92 (100)	
		Yes	0 (0)	1 (1.09)	
Emergence of general symptom (number of cases) 30 days follow up	Adenopathy	No	86 (100)	92 (100)	
		Yes	0 (0)	0 (0)	
	Fever	No	86 (100)	92 (100)	
		Yes	0 (0)	0 (0)	
	Headache	No	86 (100)	92 (100)	0.307
		Yes	0 (0)	0 (0)	
	Arthralgias	No	86 (100)	92 (100)	
		Yes	0 (0)	0 (0)	
	Malaise	No	86 (100)	92(100)	
		Yes	0 (0)	0 (0)	
	Weakness	No	86 (100)	92 (100)	
		Yes	0 (0)	0 (0)	
	Temperature		35.90 (0.50)	36.04 (0.50)	
Presence of local symptoms	1 day follow up	No	85 (97.70)	92 (98.93)	0.545
		Yes	2 (2.30)	1 (1.07)	
	2 days follow up	No	85 (96.59)	93 (98.94)	0.404
		Yes	3 (3.41)	1 (1.06)	

(number of cases)	14 days follow up	No	86 (98.85)	89 (98.89)	0.561
		Yes	1 (1.15)	1 (1.11)	
	30 days follow up	No	86 (100)	91 (98.91)	0.325
		Yes	0 (0)	1 (1.09)	
Presence of general symptoms (number of cases)	1 day follow up	No	78 (96.30)	84 (100)	0.080
		Yes	3 (3.70)	0 (0)	
	2 days follow up	No	86 (98.85)	92(100)	0.309
		Yes	1 (1.15)	0(0)	
	14 days follow up	No	88 (100)	91 (98.91)	0.314
		Yes	0 (0)	1 (1.09)	
	30 days follow up	No	86 (100)	92 (100)	
		Yes	0 (0)	0 (0)	
Increase of the brachial perimeter (mm) first dose	1 day follow up		2.56 (5.84)	3.49 (9.36)	0.457
	2 days follow up		1.86 (6.91)	3.51 (9.58)	
	14 days follow up		2.07 (11.93)	1.51 (9.10)	
	30 days follow up		2.23 (35.07)	1.31 (10.63)	
INR modification after vaccination	Mistakes of dosage	No	87 (100)	93 (100)	
		Yes	0 (0)	0 (0)	
	Intercurrent illnesses	No	87 (100)	93 (100)	
		Yes	0 (0)	0 (0)	
	Dietary changes	No	86 (98.85)	92 (98.93)	0.545
		Yes	1 (1.15)	1 (1.075)	
	Drug interactions	No	87 (100)	93 (100)	
		Yes			
**Second dose**					
Appearance of basic injuries in the area of administration of the vaccine (number of cases) 1 day follow up	Pain	No	4 (0.50)	4 (80)	0.850
		Yes	4 (0.50)	1 (20)	
	Erythema	No	8 (1)	3 (60)	0.346
		Yes	0 (0)	2 (40)	
	Swelling	No	6 (0.75)	4 (80)	0.068
		Yes	2 (0.25)	1 (20)	
	Hematoma	No	8 (100)	5 (100)	
		Yes	0 (0)	0 (0)	
	Granuloma	No	8 (100)	5 (100)	
		Yes	0 (0)	0 (0)	
	Heat	No	8 (100)	3 (60)	0.066
		Yes	0 (0)	2 (40)	
Emergence of general symptom (number of cases) 1 day follow up	Adenopathy	No	8 (100)	5 (100)	
		Yes	0 (0)	0 (0)	
	Fever	No	8 (100)	5 (100)	
		Yes	0 (0)	0 (0)	
	Headache	No	8 (100)	5 (100)	0.973
		Yes	0 (0)	0 (0)	
	Arthralgias	No	8 (100)	5 (100)	
		Yes	0 (0)	0 (0)	
	Malaise	No	8 (100)	5 (100)	0.300

		Yes	0 (0)	0 (0)	
	Weakness	No	8 (100)	5 (100)	
		Yes	0 (0)	0 (0)	
	Temperature		36.18 (0.34)	35.58 (0.77)	
Appearance of basic injuries in the area of administration of the vaccine (number of cases) 2 day follow up	Pain	No	6 (75)	2 (40)	0.924
		Yes	2 (25)	3 (60)	
	Erythema	No	8 (100)	3 (60)	0.138
		Yes	0 (0)	2 (40)	
	Swelling	No	6 (75)	3 (60)	0.033
		Yes	2 (25)	2 (40)	
	Hematoma	No	7 (100)	41 (100)	0.300
		Yes	0 (0)	0 (0)	
	Granuloma	No	8 (100)	5 (100)	
		Yes	0 (0)	0 (0)	
	Heat	No	8 (100)	3 (60)	0.066
		Yes	0 (0)	2 (40)	
Emergence of general symptom (number of cases) 2 days follow up	Adenopathy	No	8 (100)	5 (100)	0.300
		Yes	0 (0)	0 (0)	
	Fever	No	8 (100)	5 (100)	
		Yes	0 (0)	0 (0)	
	Headache	No	8 (100)	5 (100)	0.323
		Yes	0 (0)	0 (0)	
	Arthralgias	No	8 (100)	5 (100)	
		Yes	0(0)	0 (0)	
	Malaise	No	8 (100)	5 (100)	
		Yes	0(0)	0 (0)	
	Weakness	No	8 (100)	5 (100)	0.323
		Yes	0(0)	0 (0)	
	Temperature		36.06 (0.09)	35.92 (0.73)	
Appearance of basic injuries in the area of administration of the vaccine (number of cases) 14 day follow up	Pain	No	6 (75)	4 (100)	
		Yes	2(25)	0 (0)	
	Erythema	No	8 (100)	5 (100)	
		Yes	0 (0)	0 (0)	
	Swelling	No	8 (100)	5 (100)	
		Yes	0 (0)	0 (0)	
	Hematoma	No	8 (100)	5 (100)	
		Yes	0 (0)	0 (0)	
	Granuloma	No	7 (100)	4 (100)	
		Yes	0 (0)	0 (0)	
	Heat	No	8 (100)	5 (100)	
		Yes	0 (0)	0 (0)	
Emergence of general symptom (number of	Adenopathy	No	8 (100)	5 (100)	
		Yes	0 (0)	0 (0)	
	Fever	No	8 (100)	5 (100)	
		Yes	0 (0)	0 (0)	
	Headache	No	8 (100)	5 (100)	

cases) 14 days follow up		Yes	0 (0)	0 (0)	
	Arthralgias	No	8 (100)	5 (100)	
		Yes	0 (0)	0 (0)	
	Malaise	No	8 (100)	5 (100)	
		Yes	0 (0)	0 (0)	
	Weakness	No	8 (100)	5 (100)	
		Yes	0 (0)	0 (0)	
	Temperature		35.92 (0.64)	36 (0.23)	
Appearance of basic injuries in the area of administration of the vaccine (number of cases) 30 day follow up	Pain	No	8 (100)	4 (80)	0.618
		Yes	0 (0)	1(20)	
	Erythema	No	8 (100)	5 (100)	
		Yes	0 (0)	0 (0)	
	Swelling	No	8 (100)	5 (100)	
		Yes	0 (0)	0 (0)	
	Hematoma	No	8 (100)	5 (100)	
		Yes	0 (0)	0 (0)	
	Granuloma	No	8 (100)	5 (100)	0.335
		Yes	0 (0)	0 (0)	
	Heat	No	8 (100)	5 (100)	
		Yes	0 (0)	0 (0)	
Emergence of general symptom (number of cases) 30 days follow up	Adenopathy	No	8 (100)	5 (100)	
		Yes	0 (0)	0 (0)	
	Fever	No	8 (100)	5 (100)	
		Yes	0 (0)	0 (0)	
	Headache	No	8 (100)	5 (100)	
		Yes	0 (0)	0 (0)	
	Arthralgias	No	8 (100)	5 (100)	
		Yes	0 (0)	0 (0)	
	Malaise	No	8 (100)	5 (100)	
		Yes	0 (0)	0 (0)	
	Weakness	No	8 (100)	5 (100)	
		Yes	0 (0)	0 (0)	
	Temperature		36.12 (0.24)	35.74 (0.34)	
Presence of local symptoms (number of cases)	1 day follow up	No	8 (100)	5 (100)	
		Yes	0 (0)	0 (0)	
	2 days follow up	No	8 (100)	5 (100)	
		Yes	0 (0)	0 (0)	
	14 days follow up	No	8 (100)	5 (100)	
		Yes	0 (0)	0 (0)	
	30 days follow up	No	8 (100)	5 (100)	
		Yes	0 (0)	0 (0)	
Presence of general symptoms (number of cases)	1 day follow up	No	8 (100)	5 (100)	
		Yes	0 (0)	0 (0)	
	2 days follow up	No	8 (100)	5 (100)	
		Yes	0(0)	0 (0)	
		No	8 (100)	5 (100)	

	14 days follow up	Yes	0 (0)	0 (0)	
	30 days follow up	No	8 (100)	5 (100)	
		Yes	0 (0)	0 (0)	
Increase of the brachial perimeter (mm) second dose	1 day follow up		6.25 (11.88)	8 (7.58)	
	2 days follow up		5 (9.26)	8 (10.95)	
	14 days follow up		7.5 (13.89)	3 (4.47)	
	30 days follow up		8.13 (13.61)	5 (7.07)	
INR modification after vaccination	Mistakes of dosage	No	8 (100)	5 (100)	
		Yes	0 (0)	0 (0)	
	Intercurrent illnesses	No	8 (100)	5 (100)	
		Yes	0 (0)	0 (0)	
	Dietary changes	No	8 (100)	5 (100)	
		Yes	0 (0)	0 (0)	
	Drug interactions	No	8 (100)	5 (100)	
		Yes	0 (0)	0 (0)	
**Third dose**					
Appearance of basic injuries in the area of administration of the vaccine (number of cases) 1 day follow up	Pain	No	11 (68.75)	10 (62.50)	0.691
		Yes	5 (31.25)	6 (37.50)	
	Erythema	No	15 (93.75)	10 (62.50)	0.619
		Yes	1 (6.25)	6 (37.50)	
	Swelling	No	16 (100)	10 (62.50)	0.141
		Yes	0 (0)	6 (37.50)	
	Hematoma	No	15 (93.75)	16 (100)	
		Yes	1 (6.25)	0 (0)	
	Granuloma	No	16 (100)	16 (100)	
		Yes	0 (0)	0 (0)	
	Heat	No	15 (93.75)	13 (81.25)	0.308
		Yes	1 (6.25)	3 (18.75)	
Emergence of general symptom (number of cases) 1 day follow up	Adenopathy	No	16 (100)	16 (100)	
		Yes	0 (0)	0 (0)	
	Fever	No	16 (100)	16 (100)	
		Yes	0 (0)	0 (0)	
	Headache	No	16 (100)	16 (100)	
		Yes	0 (0)	0 (0)	
	Arthralgias	No	16 (100)	16 (100)	
		Yes	0 (0)	0 (0)	
	Malaise	No	16 (100)	16 (100)	
		Yes	0 (0)	0 (0)	
	Weakness	No	16 (100)	16 (100)	
		Yes	0 (0)	0 (0)	
	Temperature		35.59 (0.36)	35.91 (0.46)	

Appearance of basic injuries in the area of administration of the vaccine (number of cases) 2 day follow up	Pain	No	11 (68.75)	13 (81.25)	0.901
		Yes	5 (31.25)	3 (18.75)	
	Erythema	No	14 (87.50)	12 (75.00)	0.276
		Yes	2 (12.50)	4 (25.00)	
	Swelling	No	15 (93.75)	11 (68.75)	0.918
		Yes	1 (6.25)	5 (31.25)	
	Hematoma	No	8 (88.89)	10 (100)	
		Yes	1 (1.11)	0 (0)	
	Granuloma	No	16 (100)	16 (100)	
		Yes	0 (0)	0 (0)	
	Heat	No	15 (93.75)	13 (81.25)	0.686
		Yes	1 (6.25)	3 (18.75)	
Emergence of general symptom (number of cases) 2 days follow up	Adenopathy	No	16 (100)	16 (100)	
		Yes	0 (0)	0 (0)	
	Fever	No	16 (100)	16 (100)	
		Yes	0 (0)	0 (0)	
	Headache	No	16 (100)	16 (100)	
		Yes	0 (0)	0 (0)	
	Arthralgias	No	16 (100)	16 (100)	
		Yes	0 (0)	0 (0)	
	Malaise	No	16 (100)	16 (100)	
		Yes	0 (0)	0 (0)	
	Weakness	No	16 (100)	16 (100)	
		Yes	0 (0)	0 (0)	
	Temperature		35.80 (0.43)	35.78 (0.45)	
Appearance of basic injuries in the area of administration of the vaccine (number of cases) 14 day follow up	Pain	No	10 (62.50)	13 (81.25)	0.244
		Yes	6 (37.50)	3 (18.75)	
	Erythema	No	16 (100)	16 (100)	
		Yes	0 (0)	0 (0)	
	Swelling	No	16 (100)	14 (87.50)	
		Yes	0 (0)	2 (12.50)	
	Hematoma	No	16 (100)	16 (100)	
		Yes	0 (0)	0 (0)	
	Granuloma	No	9 (100)	9 (100)	
		Yes	0 (0)	0 (0)	
	Heat	No	16 (100)	16 (100)	
		Yes	0 (0)	0 (0)	
Emergence of general symptom (number of cases) 14 days follow up	Adenopathy	No	16 (100)	16 (100)	
		Yes	0 (0)	0 (0)	
	Fever	No	16 (100)	16 (100)	
		Yes	0 (0)	0 (0)	
	Headache	No	15 (93.75)	16 (100)	
		Yes	1 (6.25)	0 (0)	
	Arthralgias	No	16 (100)	16 (100)	
		Yes	0 (0)	0 (0)	
	Malaise	No	16 (100)	16 (100)	

		Yes	0 (0)	0 (0)
	Weakness	No	16 (100)	16 (100)
		Yes	0 (0)	0 (0)
	Temperature		35.64 (0.41)	35.73 (0.49)
Appearance of basic injuries in the area of administration of the vaccine (number of cases) 30 day follow up	Pain	No	11 (73.33)	14 (87.50)
		Yes	4 (26.67)	2 (12.50)
	Erythema	No	15 (100)	16 (100)
		Yes	0 (0)	0 (0)
	Swelling	No	15 (100)	16 (100)
		Yes	0 (0)	0 (0)
	Hematoma	No	15 (100)	16 (100)
		Yes	0 (0)	0 (0)
	Granuloma	No	15 (100)	16 (100)
		Yes	0 (0)	0 (0)
	Heat	No	15 (100)	16 (100)
		Yes	0 (0)	0 (0)
Emergence of general symptom (number of cases) 30 days follow up	Adenopathy	No	15 (100)	16 (100)
		Yes	0 (0)	0 (0)
	Fever	No	15 (100)	16 (100)
		Yes	0 (0)	0 (0)
	Headache	No	14 (93.33)	16 (100)
		Yes	1 (6.67)	0 (0)
	Arthralgias	No	15 (100)	16 (100)
		Yes	0 (0)	0 (0)
	Malaise	No	15 (100)	16 (100)
		Yes	0 (0)	0 (0)
	Weakness	No	15 (100)	16 (100)
		Yes	0 (0)	0 (0)
	Temperature		35.69 (0.41)	35.93 (0.44)
Presence of local symptoms (number of cases)	1 day follow up	No	16 (100)	16 (100)
		Yes	0 (0)	0 (0)
	2 days follow up	No	18 (100)	16 (100)
		Yes	0 (0)	0 (0)
	14 days follow up	No	15 (93.75)	16 (100)
		Yes	1 (6.25)	0 (0)
	30 days follow up	No	15 (100)	16 (100)
		Yes	0 (0)	0 (0)
Presence of general symptoms (number of cases)	1 day follow up	No	15 (100)	16 (100)
		Yes	0 (0)	0 (0)
	2 days follow up	No	16 (100)	16 (100)
		Yes	0 (0)	0 (0)
	14 days follow up	No	18 (100)	16 (100)
		Yes	0 (0)	0 (0)
Increase of the brachial perimeter	1 day follow up		0.94 (2.02)	10.94 (30.83)
	2 days follow up		4.38 (6.80)	9.06 (27.40)
	14 days follow up		6 (17.59)	4.38 (29.26)

(mm) third dose	30 days follow up		10.87 (24.72)	7.50 (30.22)	
INR modification after vaccination	Mistakes of dosage	No	16 (100)	16 (100)	
		Yes	0 (0)	0 (0)	
	Intercurrent illnesses	No	16 (100)	16 (100)	0.327
		Yes	0 (0)	0 (0)	
	Dietary changes	No	15 (93.75)	16 (100)	
		Yes	1 (6.25)	0 (0)	
	Drug interactions	No	16 (100)	16 (100)	
		Yes	0 (0)	0 (0)	

2. Figure

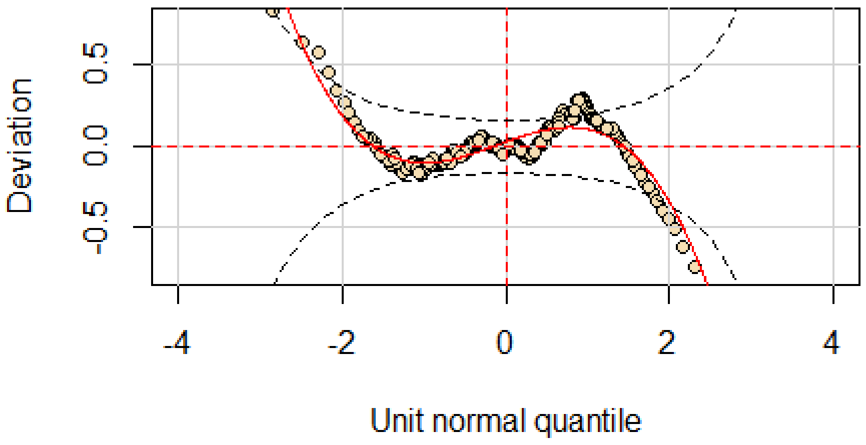

Figure 1: Representation of the gamlss model residuals
